# The Effects of the Fire Hose Square Knot Browser as a Foraging Enrichment Device on the Behavior of Captive *Macaca fascicularis*

**DOI:** 10.3390/vetsci11110535

**Published:** 2024-11-01

**Authors:** Puji Rianti, Tamara M. Anisa, Huda S. Darusman

**Affiliations:** 1 Department of Biology, IPB University, Dramaga Campus, Bogor 16680, Indonesia; tamaramuna@apps.ipb.ac.id; 2 Primate Research Centre, IPB University, Lodaya Bogor, Bogor 16151, Indonesia; hudada@apps.ipb.ac.id; 3 School of Veterinary and Biomedical, IPB University, Dramaga Campus, Bogor 16680, Indonesia

**Keywords:** animal welfare, breeding facilities, cynomolgus macaques, daily behavior, enclosure enrichment tools

## Abstract

Effective management of captive *M. fascicularis* (long-tailed macaques) is crucial for maintaining high-quality research models, necessitating strategies to promote their welfare. This study evaluated the impact of a foraging enrichment device, the “fire hose square knot browser”, on the behavior of 32 long-tailed macaques at the Primate Research Center of IPB University in Bogor, Indonesia. We observed and analyzed daily behaviors across various food types over 288 h using scan and instantaneous sampling methods. Statistical analyses, including ANOVA and Kruskal–Wallis tests, revealed significant behavioral changes in the presence of the foraging enrichment device. The foraging enrichment device notably increased eating and affiliative behaviors, while reducing resting, agonistic, and stereotypic behaviors. No significant differences in eating, auto-grooming, resting, sexual, agonistic, or stereotypic behaviors were observed between the morning and afternoon. However, mobility and affiliation behaviors varied between these periods. The highest percentage of eating behavior occurred with the enrichment foraging device and a mixture of fruits. Furthermore, a three-way non-parametric analysis suggests a significant effect of food type and treatment on behaviors such as mobility and stereotyping. These findings underscore the positive impact of foraging enrichment devices on promoting active behavior and enhancing animal welfare in captive long-tailed macaques.

## Simple Summary

Interdisciplinary studies on animal behavior and health management have increased over the years. Interdisciplinary collaboration involving behaviorists and veterinarians is vital for advancing animal welfare, particularly in diverse settings, such as farms, zoos, and laboratories. Behaviorists design enrichment devices to promote species-typical behaviors, while veterinarians ensure that these devices are safe to balance natural behaviors with safety, particularly for non-human primates in captivity such as *Macaca fascicularis* (long-tailed macaques). One way to achieve this is by adding an enrichment device, such as a fire hose square knot browser, to stimulate natural behaviors, improve welfare, and reduce stress-related behaviors. Here, we examined the impact of the device on the behavior of 32 captive long-tailed macaques using scanning and instantaneous sampling methods. We found that the device significantly increased eating and affiliative behaviors, while reducing resting, agonistic, and stereotypic behaviors. These findings suggest that the fire hose square knot browser effectively promotes natural foraging behaviors and enhances social interactions of captive long-tailed macaques. The study highlights the importance of these devices in managing and aligning with animal welfare science, emphasizing interdisciplinary collaboration and implementing strategies that ensure the physical and mental well-being of animals in captive facilities.

## 1. Introduction

Recent research in animal welfare has increasingly emphasized the importance of practical collaborations between behaviorists and veterinarians to enhance the care and management of captive animals, particularly in breeding facilities [1,2,3,4]. Behaviorists focus on designing enrichment devices that stimulate natural behaviors, encouraging problem-solving and exploration critical to species-typical behaviors, and play a vital role in this process. Their work aimed to promote positive behaviors, such as foraging and social interactions, and to reduce negative behaviors, such as resting, agonistic, and stereotypic behavior. These negative behaviors are often linked to stress and poor welfare conditions in captivity [5,6]. However, veterinarians ensure that these devices are safe and do not pose any health risks to animals. They assess the physical well-being of animals and monitor any changes in health status resulting from the introduction of new enrichment devices [7]. Enrichment devices can significantly decrease the incidence of injuries caused by conflicts and repetitive stress by reducing agonistic and stereotypic behaviors [6,8]. Enrichment devices enhance the overall veterinary care program by minimizing the need for medical interventions and improving the quality of life of animals [9,10,11]. This partnership is critical for developing and implementing enrichment strategies that promote natural behavior while ensuring the safety and health of animals. The daily behavior of long-tailed macaques living in captivity may differ from that observed in their natural habitats. However, in breeding facilities that prioritize animal welfare, the daily behavior of long-tailed macaques often exhibits similar activity patterns across habitats [12].

**Figure 1:**
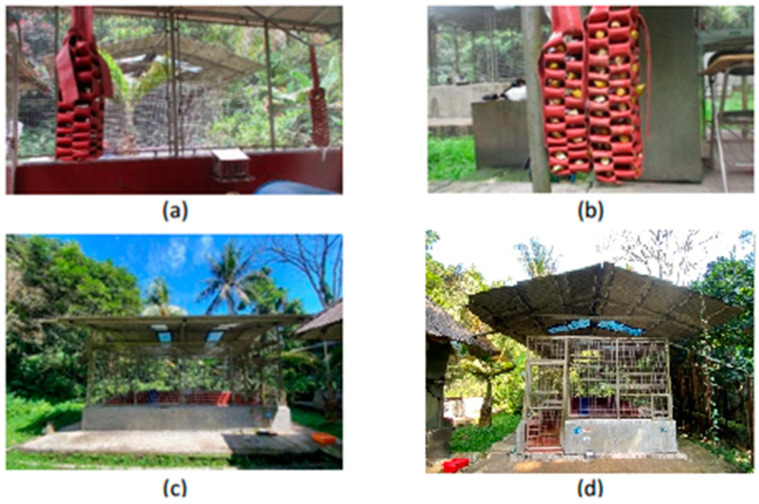
The fire hose knot browser (foraging enrichment device) and enclosures. Panel (**a**) is the empty foraging enrichment device, while panel (**b**) is the filled-in foraging enrichment device. Panels (**c**,**d**) represent the enclosures K1 and K2, respectively.

While foraging enrichment devices are common in many settings [13,14,15,16], their use in Indonesian breeding facilities is still emerging. This study provides essential data on the efficacy of the fire hose square knot browser as an enrichment tool within this context. The device, designed to stimulate natural foraging behaviors, addresses key welfare concerns by promoting problem solving, exploration, and reducing stress-related behaviors such as stereotypies. Notably, the behavioral benefits of this device were assessed along with veterinary reviews of its safety, ensuring a holistic approach to enrichment. In this study, we examined the introduction of a specific enrichment device, the fire hose square knot browser, into a breeding facility (see Figure 1a,b) housing *Macaca fascicularis* (long-tailed macaques) and assessed its impact on both behavioral outcomes and overall welfare. This study highlights how the fire-hose square knot browser can serve as a model for integrating these two aspects to improve the welfare of captive macaques in breeding facilities.

We focus on how the device influences foraging behavior, social interactions, and stress reduction in long-tailed macaques that are commonly used in breeding and research facilities. By conducting a detailed evaluation of the impact of the device on behavior and welfare, we aim to contribute to the best practices for environmental enrichment in breeding centers. This research offers insights into the specific behavioral outcomes of introducing this type of enrichment, while also addressing safety concerns central to veterinary care. The findings from this study will not only advance the understanding of how foraging devices such as the fire hose square knot browser can benefit the welfare of long-tailed macaques but also provide guidance for breeding facilities looking to adopt more effective and safe enrichment strategies. Ultimately, this study underscores the value of interdisciplinary collaboration in enhancing animal welfare, aligning with the goals of this special issue to showcase the impact of behaviorist-veterinary partnerships.

## 2. Materials and Methods

Our study was conducted simultaneously with the macaque breeding program, daily behavior activities, and feeding schedules following the captive management protocols of long-tailed macaque breeding facilities at the Primate Research Center, IPB University, West Java, Indonesia (PRC-IPB). The Animal Care and Use Committee of Ethics (ACUC-IPB University) approved this study (permit no. IPB PRC-22-A006).

### 2.1. Animals and Enclosures

We observed 32 long-tailed macaques housed in two enclosed environments within an animal biosafety level 1 facility. Enclosure 1 (K1) housed 19 individuals, comprising one adult male, 16 adult females, and two juveniles, whereas enclosure 2 (K2) housed 13 individuals, including one adult male, 11 adult females, and one juvenile. All long-tailed macaques ranging in age from 1 to 10 years were confirmed as F1 breeds, and were born and bred within the facilities of PRC-IPB. Each LTM was assigned a unique identification number and underwent a daily health check-up by the attending veterinarian in the facility (only average body weight was used in this study).

Each enclosure was 6 m in length, 5 m in width, and 2.5 m in height, with a distance of 4 m between them. Both enclosures featured stainless-steel wire walls on all four sides, allowing long-tailed macaques to observe their surroundings (see Figure 1c,d). The keeper cleaned the enclosures daily at 07:00.

### 2.2. Design and Procedures

We used two fire hose square knot browsers as additional foraging enrichment devices in the enclosures, which served as observational devices in this study. These devices were suspended inside breeding enclosures, designated as K1. These devices aim to provide an alternative food source to enhance social engagement and encourage positive active behavior as a manifestation of natural behavior within long-tailed macaque groups housed in breeding enclosures. The fire hose square knot browser is constructed in the shape of a pocket made from a 0.04-m thick and 15-m-long fire hose. The resulting product measured approximately 1 m in length and featured 72 and 88 holes, respectively, for dispensing feed (see Figure 1a,b). The devices were approved by the ACUC-IPB University for use in this experiment, implying that they are safe to use from the health perspectives of veterinary and breeding facilities.

We conducted a habituation phase with the study participants during the initial 14 days of the experiment. This phase acclimated long-tailed macaques to the presence of the researchers, desensitizing them to their presence before the onset of the study. Subsequently, we allocated two enclosures for a conditioning period of 24 days, encompassing two control treatments and treatments involving the presence or absence of additional fire-hose devices. The control conditions, designated as C1 (in K1) and C2 (in K2), did not include fire hose devices in their respective enclosures during the initial 1-6 days. The treatment involving foraging enrichment devices (E) involved introducing fire hose square knot browser devices to the enclosure (in K1) from day 7 to the 12th day.

**Figure 2:**
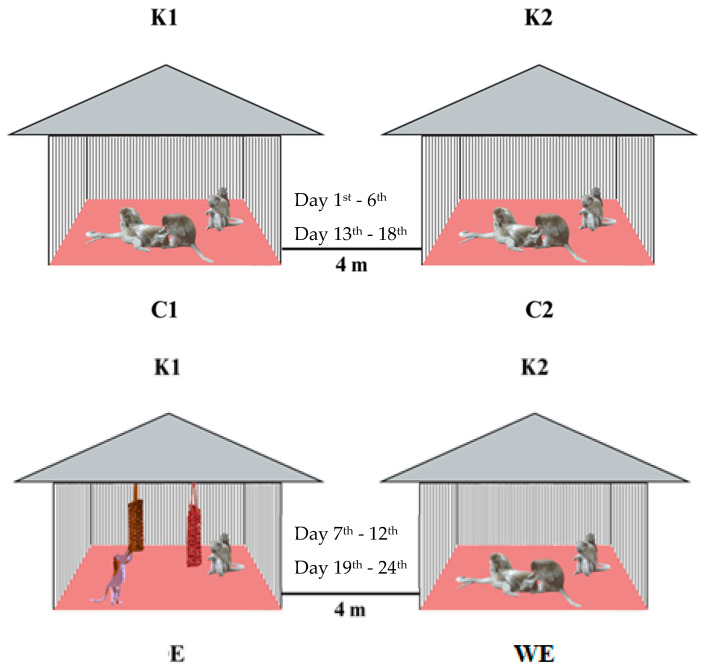
The two control conditions (**C1**, **C2**), the presence of enrichment device (E), and the absence of the device (without device; WE) of a foraging enrichment device in the enclosures (**K1**, **K2**). The observed study is indicated during the days described between enclosures. The hanging fire hose knot browser (foraging enrichment device) is shown inside (**K1**) in the presence of device conditions.

Additionally, we conditioned the long-tailed macaques at K2 to observe the environmental conditions of K1 during the same period (7th–12th), wherein K1 underwent fire hose treatment. This condition at K2, coinciding with the display of E in K1, was termed without treatment (WE). We conducted this WE experiment to determine whether a behavioral effect resulted from a group of long-tailed macaques observing their neighbors having a new “toy” as a foraging enrichment device in the enclosure. These experiments were replicated on subsequent days, from the 13th to the 18th for C1 and C2 and from the 19th to the 24th for E and WE, respectively (see Table 1 and Figure 2).

Each group of long-tailed macaques in both enclosures (K1 and K2) received various types or combinations of food, according to the daily feeding protocols established by captive management at PRC-IPB. The diet included commercial local and Bangkok monkey chow, supplied by PT Citra Ina Feedmill and Good Pet, Charoen Pokphand, Thailand, bananas, guavas, and yams, respectively (Table 1). PRC-IPB management determines whether to utilize local or Bangkok monkey chow, or a blend of both in the diet.

In the enclosures during C1, C2, and WE, fruits are distributed by tossing them onto the top side of the enclosure to scatter them on the enclosure floor. Additionally, fruits and/or monkey chow were provided during the treatment of the foraging enrichment device (E) by inserting them into the holes of the fire hose square knot browser. Juvenile and adult long-tailed macaques received 50 g of diet per eating period, while lactating female long-tailed macaques received 60 g. Further details regarding the diet comparisons during the treatment study are presented in Table 1.

### 2.3. Daily Behavior Activities Records

We used a combination of scan and instantaneous sampling methods [17] to observe the daily behavior of long-tailed macaques. Scan sampling was conducted during two observation periods, from 08:00 to 11:00 h and from 13:00 to 16:00 h, to broadly assess the behavior of macaques. Instantaneous sampling was then employed at 5 min intervals during the same observation periods to record the presence or absence of specific behaviors. Food was provided to the macaques at 08:00 and 13:00. The behaviors recorded included food-related behaviors (eating), solitary behaviors (resting, auto-grooming, mobility, and stereotypic behaviors), and social behaviors (affiliative, sexual, and agonistic behaviors). A detailed ethogram with modifications is provided in Supplementary Material, Table S1.

### 2.4. Statistical Analysis

We systematically recorded the frequency and percentage of daily behavioral activities of long-tailed macaques in K1 and K2 over 288 h of observation, spanning a total of 24 days. The data encompassed observations during control conditions (C1 and C2), treatments involving the introduction of a foraging enrichment device (E), and treatments without devices (WE). Activity percentages were analyzed using the following formula: activity frequency divided by the total frequency of behavior activities × 100% of the total frequency of behavior activities [18]. Subsequently, we compared the treatments and observation periods with regard to the daily behavioral activities of long-tailed macaques. Additionally, we compared the effects of different diet types and the presence (E) or absence (WE) of additional foraging enrichment devices on the eating behavior of long-tailed macaques. We used the Analysis of Variance (ANOVA) or Kruskal–Wallis and Mann–Whitney tests at a significance level of *p* < 0.05. In cases where a significant difference was detected in the ANOVA, we proceeded with Duncan’s multiple range test (DMRT) for further analysis to measure specific differences between pairs of means. We also performed a three-way ANOVA or the Kruskal–Wallis and Mann–Whitney tests for time, feeding treatment, and food type parameters at a significance level of *p* < 0.05 for each kind of behavior to analyze whether the device has an impact on behavior for all foods or just some of them. All analyses were conducted using the R Studio software version 4.2.3 [19].

## 3. Results

### 3.1. Daily Behavior Activities and Body Weight of Long-Tailed Macaques in Breeding Facilities

**Figure 3:**
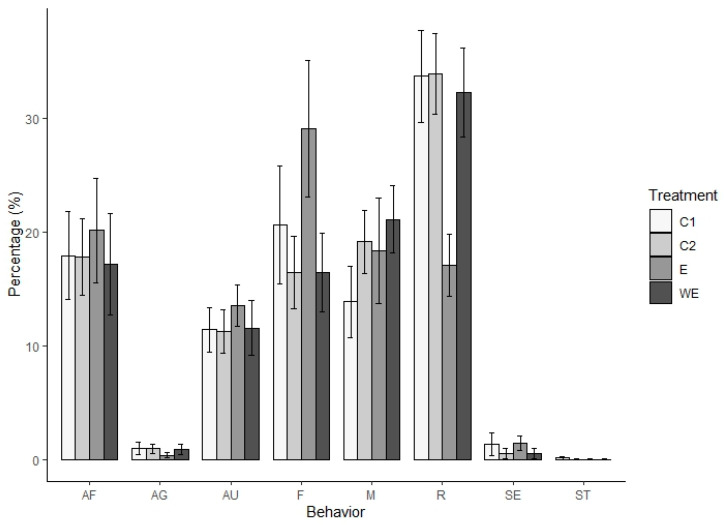
The percentage of daily behaviors exhibited by *Macaca fascicularis* across four conditions. C1 = the control condition in enclosure 1 and C2 = the control condition in enclosure 2. E = the treatment of foraging enrichment devices in enclosure 1, and WE = the absence of treatment foraging enrichment devices in enclosure 2. The behaviors are categorized as follows: AF = affiliating behavior, AG = agonistic behavior, AU = auto-grooming behavior, F = eating behavior, M = mobility behavior, R = resting behavior, SE = sexual behavior, and ST = stereotyping behavior.

The implementation of treatment in this study resulted in varying percentages of daily behavioral activities among long-tailed macaques. Affiliating, auto-grooming, and feeding behaviors were the three most frequently observed activities in the presence of the foraging enrichment devices (E in K1), whereas moving behaviors were highest in the groups without a foraging enrichment device (WE in K2). The addition of the foraging enrichment treatment to K1 reduced the percentage of resting, agonistic, and stereotypical behavior of long-tailed macaques. However, without the treatment of foraging enrichment devices at K2, affiliative, auto-grooming, and feeding behavior showed a lower score than E, similar to the control conditions (C1 and C2). In contrast, in moving behavior, WE scored the highest (see Figure 3).

The ANOVA test showed significant differences in the resting (*p* = 2 × 10^−16^), affiliating (*p* = 0.041), and auto-grooming (*p* = 4 × 10^−4^) behavior of long-tailed macaques across the two control conditions (C1 and C2) and the treatment involving the presence (E) and absence (WE) of foraging enrichment devices. Subsequently, these results were further examined using DMRT to evaluate the differences between all conditions for these three behaviors. The DMRT findings indicated significant differences exclusively in resting, affiliating, and auto-grooming behaviors when foraging enrichment devices were present (E) (refer to the detailed DMRT values in Table 2).

The presence of the foraging enrichment device treatment (E) had the highest median percentage of affiliating behavior (including auto-grooming) compared to the two control conditions and the absence of foraging enrichment devices (see Figure 3). Furthermore, the study revealed a correlation between the effect of the two control conditions and the two treatments with the observation period on the percentage of long-tailed macaque resting behavior (*p* = 0.048) and affiliating behavior (*p* = 0.043), but not in auto-grooming behavior (*p* = 0.236). Significant differences were also observed between the morning and afternoon observation periods for affiliating behaviors (*p* = 0.001). However, resting and auto-grooming showed no significant difference between the two observation periods (*p* = 0.659 and *p* = 0.293, respectively) (for details, please see Supplementary Materials, Figure S1).

Moreover, given the non-normally distributed data (see Supplementary Materials, Analysis Set S1) on eating, mobility, sexual, agonistic, and stereotypic behavior, we conducted analyses using the Kruskal–Wallis test to assess the significance of behavior across two control conditions and two treatments. The Kruskal–Wallis test showed significant differences in eating (*p* = 2.660 × 10^−11^), mobility (*p* = 6.571 × 10^−9^), sexual (*p* = 7.308 × 10^−9^), agonistic (*p* = 4.831 × 10^−7^), and stereotypic (*p* = 4.209 × 10^−6^) behaviors of long-tailed macaques across the two control conditions and two treatments. Only mobility behavior (*p* = 3 × 10^−3^) showed a significant difference between the two time periods, while eating, sexual, agonistic, and stereotypic behaviors showed no significant difference between the morning and afternoon data (*p* = 0.570, *p* = 0.056, *p* = 0.057, and *p* = 0.299, respectively; see Supplementary Materials, Figure S1).

Additionally, based on the health record data before and after the experiment (see Supplementary Materials, Data Set S1; Analysis Set S2), there was an increase in body weight in both cages. The normality tests of the body weight data for K1 were non-normally distributed. Therefore, we used the Mann–Whitney test to observe a significant difference in body weight before and after the foraging enrichment devices. Meanwhile, the long-tailed macaque group at K2 showed no significant difference in body weight during the one-way ANOVA experiment. Hence, we can assume that the presence of a fire hose knot browser influences the increase in body weight of long-tailed macaques.

### 3.2. The Association Between the Types of Diets Administered During the Study and Type of Behaviors Exhibited by Long-Tailed Macaques

We then examined eating behavior in detail during the two control conditions (C1 and C2) and two treatments (E and WE). Serving a mixture of Bangkok monkey chow and yam resulted in the highest percentage of eating behavior compared to serving Bangkok monkey chow alone or in other diet combinations with the presence of foraging enrichment devices (Supplementary Materials, Figure S2).

**Figure 4:**
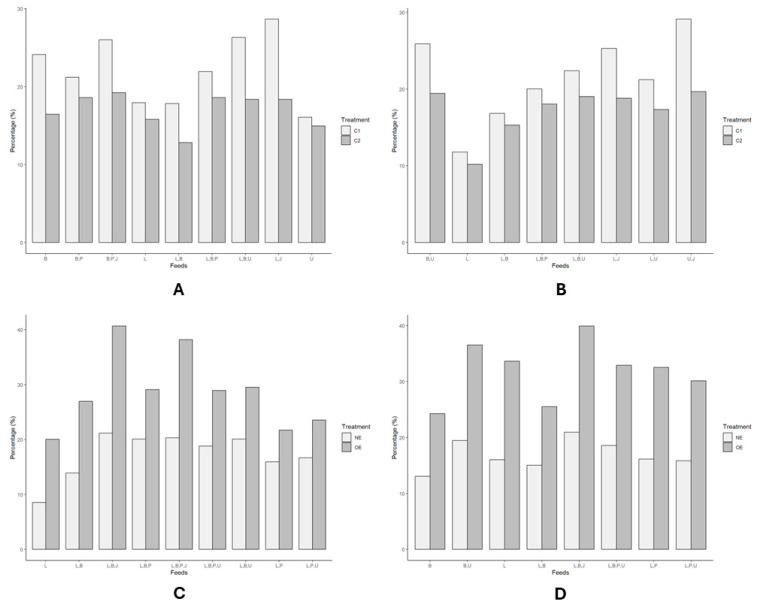
The eating behavior was compared across four conditions, two periods of observation, and various types of diets in *Macaca fascicularis*. C1 = the control treatment in enclosure 1, and C2 = the control treatment in enclosure 2. E = the foraging enrichment devices in enclosure 1, and WE = the absence of foraging enrichment devices in enclosure 2. Feeds represent types of diets. (**A**) = the two control conditions in enclosures 1 and 2 during the morning, and (**B**) = the two control conditions in the afternoon. (**C**) = both the presence and absence of foraging enrichment devices in the morning and (**D**) = the same treatment in the afternoon. The types of diets include Bangkok monkey chow (B), local monkey chow (L), banana (P), and yam (U).

The Mann–Whitney test showed no significant difference between the two control conditions (C1 and C2) in the morning (*p* = 0.112), but a significant difference was observed in the afternoon (*p* = 0.010). However, in the presence (E) and absence (WE) of foraging treatment devices, the Mann–Whitney test indicated significant differences both in the morning (*p* = 9.667 × 10^−5^) and afternoon (*p* = 7.396 × 10^−7^). Additionally, the analysis demonstrated a significant difference between the presence and absence of treatment food devices concerning the type of diet diversity (*p* = 2.263 × 10^−5^) (see Figure 4). This finding suggests that adding a fire hose square knot browser as a foraging enrichment device increases the percentage of LTM eating behavior.

In addition, we examined the three-way ANOVA to predict the impact of time, treatment, and type of diet on each type of behavior. The analysis showed no significant differences between time, treatment, and type of diet in eating, resting, affiliate, auto-grooming, sexual, and aggressive behaviors. Meanwhile, owing to the non-normally distributed data on mobility and stereotyping behavior, we performed the Kruskal–Wallis non-parametric test for the impact of time, treatment, and type of diet. The analysis showed a significant difference in time, treatment, and diet for mobility and stereotyping behavior (please refer to Supplementary Materials, Analysis Set S3). This finding indicates that feeding enrichment devices significantly influence mobility and stereotyping behavior in all types of diets.

## 4. Discussion

Implementing fire hose square knot browsers as foraging enrichment devices in the breeding enclosures of long-tailed macaques in PRC-IPB significantly affected their daily behavior. Foraging enrichment devices are particularly beneficial for captive primates because they enhance their welfare by promoting species-appropriate behaviors [14]. This study represents the first examination of how these types of foraging enrichment devices affect the daily behavior of long-tailed macaques, particularly in a breeding facility at PRC-IPB, Indonesia. However, it is important to recognize that this study primarily focused on behavioral outcomes, and veterinary considerations such as animal conditioning, reproductive status, and potential injury should not be overlooked. While enrichment devices successfully increased affiliating, foraging, and exploratory behaviors, it is equally vital to acknowledge how such devices can influence broader health and veterinary care aspects, particularly in ex situ or breeding facility management.

From a veterinary perspective, one key observation was that there were no injuries or adverse physical health outcomes in the experiment. Moreover, we observed an increase in the body weight during the treatment period (K1). This suggests that the presence of the fire hose knot browser positively influenced macaque body weight, likely due to the increased foraging and feeding behaviors facilitated by the enrichment device [20]. This is an important finding, as it indicates that the foraging enrichment devices used in this study did not pose any significant risk to the physical well-being of macaques. Additionally, the increase in affiliating behaviors, including social grooming and positive social interactions, highlights the role of enrichment in fostering a harmonious environment that may prevent stress-related injuries caused by aggression or social tension. The observed reduction in agonistic behaviors further supports this statement, as less aggression likely reduces the incidence of injuries within a group [20,21].

Macaques housed in captivity are often accustomed to being fed in food trays without any training in foraging or independently obtaining food. The fire hose square knot browser was used as a modified feeding bin for long-tailed macaques to engage in foraging and food search activities. Consequently, long-tailed macaques have more opportunities to extract visible and hidden food from their foraging enrichment devices. Enrichment techniques are designed to encourage captive primates to engage in species-appropriate foraging behaviors, as they often spend less time searching for, obtaining, and processing food than their wild counterparts [20].

Our findings regarding daily behavior with foraging enrichment devices revealed a heightened frequency of eating and affiliating behaviors, including allo-grooming, and reduced resting, agonistic, and stereotypic behaviors among long-tailed macaques. These results align with previous studies examining foraging racks and shavings as foraging enrichment devices in *Macaca mulatta*, which demonstrated increased activity alongside decreased agonistic behavior [22]. The broader array of foraging devices available to long-tailed macaques results in a greater diversity of foraging sources in captivity [23], leading to increased foraging time and decreased resting and agonistic behavior.

Moreover, our observations indicate that long-tailed macaques’ affiliating and mobility behavior did not differ between the morning and afternoon periods, whereas other behaviors did. Interestingly, long-tailed macaques exhibited increased eating behavior in the afternoon compared to that in the morning. This pattern may be attributed to the higher activity levels observed in the morning, such as affiliating and sexual behavior, resulting in a greater need for energy before settling in the evening [24]. The feeding time of 12:30 h marks the last feeding opportunity for the day. This behavior mirrors patterns observed in their wild counterparts, specifically in the Telaga Warna Nature Reserve [25].

Implementing the fire hose square knot browser as a foraging enrichment device successfully extended the period when captive groups of long-tailed macaques engaged in food search and acquisition activities. Providing foraging food enrichment devices reduces undesirable behaviors and increases positive natural animal behaviors [26]. Our results indicate that foraging enrichment devices significantly influence eating behavior compared with conditions without such devices. However, it is essential to note that the type of food can also strongly affect eating behavior in captive long-tailed macaques [27]. Our observations revealed that captive long-tailed macaques showed a preference for and consumed fruit diets over monkey chows (Bangkok and local), consistent with findings in various LTM captive groups [28,29] and in Barbary macaques [30]. Fruits are known to contain more complex macronutrients than instant monkey chow, which is also a highly processed food. Overall, the total energy used by monkey chow is 0.38 Kcal, while fruits range between 79–98 Kcal per 100 g served diets [31].

Our study demonstrated increased foraging and locomotion behavior when the fire hose square knot browser was added, indicating increased exploration of foraging enrichment devices for food and reduced resting behavior [27]. Fire hose square knot browsers showed increased affiliating behaviors, such as allo-grooming and sexual behaviors [32]. Allo-grooming among long-tailed macaques strengthens social relationships [33,34], which may be necessary for cooperation in obtaining food from foraging enrichment devices [27,30]. Additionally, allo-grooming can serve as a form of reconciliation or conflict avoidance, and as a positive approach to females before copulation [35]. This behavior remained significant in groups of long-tailed macaques with a high number of individuals (one male with 16 females and two juveniles). We observed an increase in sexual behavior in the presence of foraging enrichment devices, although it constituted a deficient percentage of the daily activity budget compared to all behaviors. In this comfortable environment, they may engage in more positive affiliative behaviors or mating [22].

We also considered that the presence of foraging enrichment devices reduced or did not cause stress in the long-tailed macaque groups at PRC-IPB. We noted that the observed stereotypical behavior showed the lowest percentage compared to the other behaviors. Animals with stereotypical behavior accounting for less than 3% of their total daily activity are generally considered at risk [36]. Additionally, we observed a slight increase in auto-grooming behavior in the presence of foraging enrichment devices, although it remained lower than allo-grooming behavior. This finding contradicts previous studies that showed low auto-grooming behavior in the presence of foraging enrichment devices owing to increased foraging or active behavior in obtaining food [20,32]. As mentioned earlier, this discrepancy may be because the long-tailed macaque group with foraging enrichment devices experienced reduced competition over food and lower social pressure (such as the absence of agonistic behavior in the enclosure), allowing them more time for self-maintenance.

Although this study presents valuable findings, some limitations must be acknowledged. The relatively short observation period may have limited the capture of long-term effects on behavior and health. Additionally, the study was conducted in a breeding group with a single male, which may not fully reflect the dynamics of multimale groups or other group compositions. Future studies should explore these variables over extended periods and in different social contexts to gain a more comprehensive understanding of the impact of enrichment devices on both behavioral and veterinary outcomes.

As expected, our findings indicate that daily behaviors with foraging enrichment devices improve positive activities and reduce negative behaviors, such as agonistic and stereotypic behaviors. This finding suggests that adding enriched food devices enhances animal welfare, particularly in terms of freedom from hunger, thirst, and malnutrition, as well as the freedom to express natural behaviors and alleviate discomfort, fear, and stress. Additionally, it addresses the domains of environment, behavior, and mental state, which are crucial for the nutritional and health achievements of long-tailed macaques. The results of this study are expected to serve as a valuable reference for the development of animal welfare management in PRC-IPB.

## 5. Conclusions

The implementation of fire hose square knot browsers as foraging enrichment devices in the breeding enclosures of long-tailed macaques at PRC-IPB significantly affected their daily behavior, improving their welfare by promoting species-appropriate behaviors. However, it is important to recognize that this study primarily focused on behavioral outcomes, and veterinary considerations such as animal conditioning, reproductive status, and potential injury should not be overlooked. While enrichment devices successfully increased affiliating, foraging, and exploratory behaviors, it is equally vital to acknowledge how such devices can influence broader health and veterinary care aspects, including injury prevention, physical conditioning, and breeding potential.

The addition of fire hose square knot browsers as foraging enrichment devices significantly influenced the daily behavior of long-tailed macaques. All behaviors exhibited significant differences between the two control conditions and treatments. Eating, affiliating, and mobility were the most frequently observed behaviors, while foraging enrichment devices significantly reduced resting, agonistic, and stereotypic behaviors. Only affiliating and mobility behaviors showed no significant differences between the morning and afternoon observation periods. Furthermore, despite the various types of food served during the observation periods, the presence of foraging enrichment devices increased eating behavior compared with the absence of these devices. These findings demonstrate that the addition of fire-hose square knot browsers influences the animal welfare of captive LTM groups in the PRC-IPB.

## Figures and Tables

**Table 1:** Design of treatments, feeding times, and diet types used in the study.

Days	Morning Feeding Time (07:30 h)				Afternoon Feeding Time (12:30 h)			
	Treatments				Treatments			
	C1	E	C2	WE	C1	E	C2	WE
1	B, U		B, U		L, B, P		L, B, P	
2	U, J		U, J		L, J		L, J	
3	L, J		L, J		L		L	
4	L		L		L		L	
5	L		L		L		L	
6	L, U		L, U		U		U	
7		L, P, U		L, P, U		L, P, U		L, P, U
8		L, P		L, P		L, P		L, P
9		L, P		L, P		L, P		L, P
10		L, B		L, B		L, B, P, U		L, B, P, U
11		L		L		L		L
12		L, B		L, B		L, B		L, B
13	L, B		L, B		B		B	
14	L, B		L, B		L, B, U		L, B, U	
15	L, B, U		L, B, U		L, B, U		L, B, U	
16	L, B, P		L, B, P		B, P		B, P	
17	L		L		B, P, J		B, P, J	
18	L, B, P		L, B, P		L, B		L, B	
19		L, B, P, J		L, B, P, J		L, B, J		L, B, J
20		L, B, J		L, B, J		L, B		L, B
21		L, B, U		L, B, U		B, U		B, U
22		L, B, P, U		L, B, P, U		L, B, P, U		L, B, P, U
13		L, B		L, B		L, B		L, B
24		L, B, P		L, B, P		B		B

Note: (C1) Control 1 in Enclosure 1, (C2) Control 2 in Enclosure 2, (E) presence of foraging enrichment devices in Enclosure 1, and (WE) absence of foraging enrichment devices in Enclosure 2. B, Bangkok monkey chow; L, local monkey chow; U, yam; J, guava; P, banana. The percentage of total feeding types is as follows: B, U (50%, 50%); U, J ((50%, 50%); L, J (50%, 50%); L (100%); L, U (50%, 50%); L, B, P (20%: 20%: 60%); L, P, U (20%: 20%: 60%); L, P (50%, 50%); L, B (50%, 50%); L, B, P, U (20%: 20%: 30%: 30%); L, B, U (20%: 20%: 60%); B, P (50%, 50%); B, P, J (20%: 20%: 60%); L, B, P, J (20%: 20%: 30%: 30%); L, B, J (20%: 20%: 60%); B (100%), each percentage corresponding to the abbreviation, respectively. The empty light grey boxes represent no data observed at that time.

**Table 2:** DMRT test of treatment parameters on *Macaca fascicularis* resting, affiliative, and auto-grooming behavior.

Treatments	Percentage of Behavior (%)		
	Rest	Affiliative	Auto-Grooming
Control 1	33.705 ^a^	17.934 ^b^	11.410 ^b^
Control 2	33.894 ^a^	17.779 ^b^	11.245 ^b^
Enrichment	17.044 ^b^	20.163 ^a^	13.529 ^a^
Without enrichment	32.265 ^a^	17.183 ^b^	11.556 ^b^

Note: Values in columns with different superscript letters are significantly different (Î ± = 0.05).

## Data Availability

Data supporting the reported results are available to the public upon request from the corresponding author.

## Supplementary Materials

The following supporting information can be downloaded from: https://www.mdpi.com/article/10.3390/vetsci11110535/s1. Figure S1: The percentage of conditions during each feeding time and the daily behavior of long-tailed macaques; Figure S2: The comparison of the eating behavior across various types of diets in long-tailed macaques under different treatments; Table S1: The ethogram of the observed daily behavior used in the study [28] with modification. Analysis Set S1: Normality test for each behavioral data point. Data Set S1: Body weight data of long-tailed macaques before and after the study. Analysis Set S2: Normality test, Mann–Whitney test, and ANOVA analysis of the body weight of long-tailed macaques. Analysis Set S3: Three-way ANOVA and Kruskal–Wallis test for time, treatment, and diet type.
