# Supplementary material for: The Effects of the Fire Hose Square Knot Browser as a Foraging Enrichment Device on the Behavior of Captive Macaca fascicularis
Source: Vet Sci. 2024 Nov 1;11(11):535. doi: 10.3390/vetsci11110535 (PMC11598876; doi:10.3390/vetsci11110535)
Supplement: Supplementary file 1 [file vetsci-11-00535-s001.zip › vetsci-3199058-supplementary.pdf]

Supplementary materials:

The effects of the fire hose square knot browser as a foraging enrichment device on the behavior of captive *Macaca fascicularis*

Puji Rianti [1,2,*], Tamara M. Anisa [1] and Huda S. Darusman [2,3]

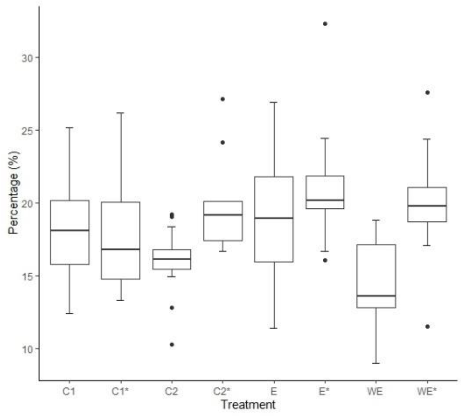

a

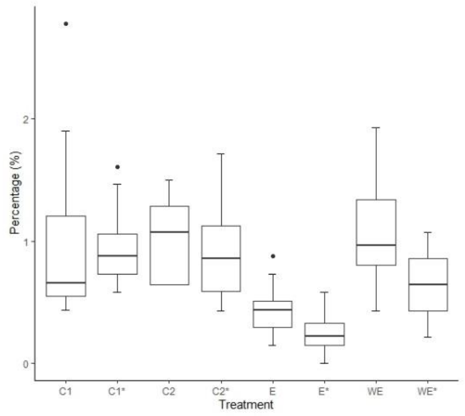

b

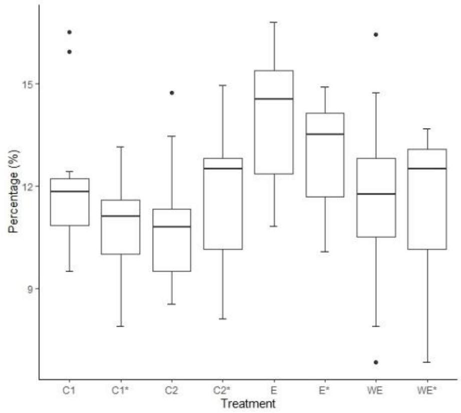

c

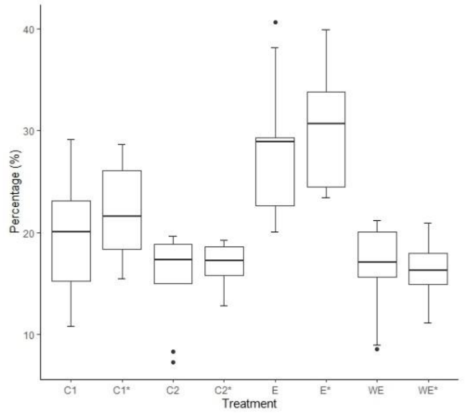

d

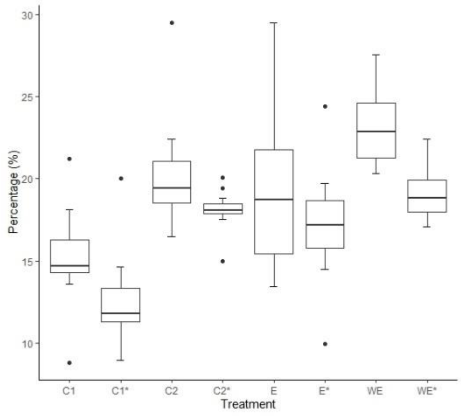

e

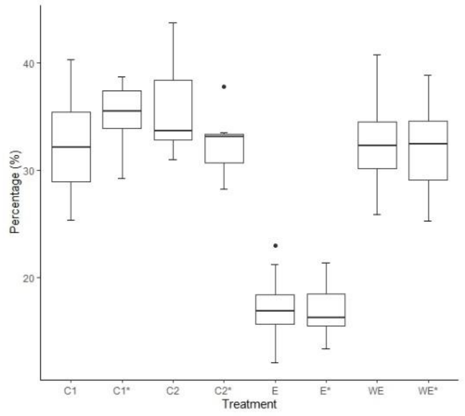

f

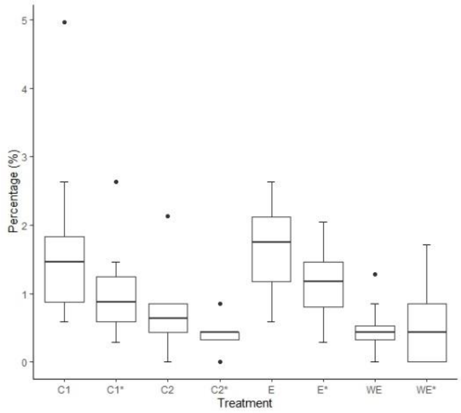

g

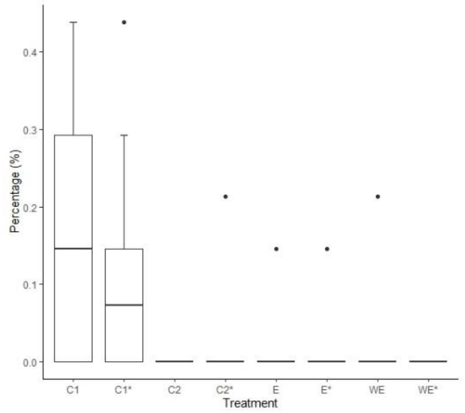

h

Figure S1. The percentage of conditions during each feeding time and the daily behavior of long-tailed macaques. C1 = the control condition in enclosure 1, and C2 = the control condition in enclosure 2. E = the foraging enrichment devices in enclosure 1, and WE = the absence of foraging enrichment devices

in enclosure 2. (*) denotes behavior in the afternoon. (**a**) affiliative behavior, (**b**) agonistic behavior, (**c**) auto-grooming behavior, (**d**) eating behavior, (**e**) moving behavior, (**f**) resting behavior, (**g**) sexual behavior, and (**h**) stereotypic behavior.

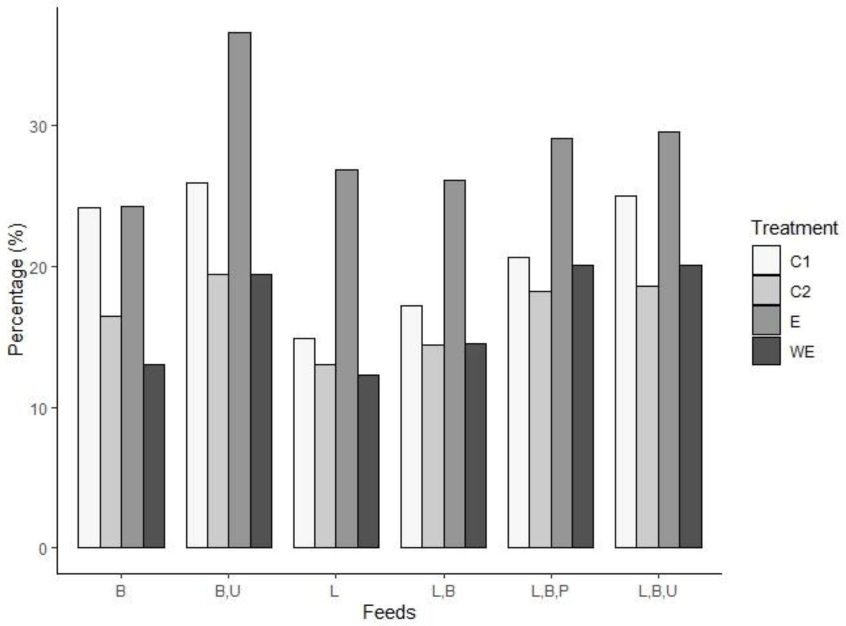

Figure S2. The comparison of the eating behavior across various types of diets in long-tailed macaques under different treatments. C1 = the control treatment in enclosure 1, and C2 = the control treatment in enclosure 2. E = the foraging enrichment devices in enclosure 1, and WE = the absence of foraging enrichment devices in enclosure 2. The types of diets correspond to different types of diets. B = Bangkok monkey chow, L = Local monkey chow, P = banana, and U = yam.

Table S1. The ethogram of the observed daily behavior used in the study [28] with modification.

Type of behavior	Behavior	Code	Description of behavior
Food-related	Eating	F	Individuals ingest food items through chewing, swallowing, and digesting the food from its environment.
Solitary	Resting	I	The individual is lying Down (adopting a recumbent posture with the body supported by a surface such as the ground, a rope, or a stand). It may occur in various positions, including ventral, dorsal, or lateral.<br>Sitting (assuming a seated posture with the body supported by the buttocks or lower limbs while remaining upright). It may involve resting on the ground or a stable surface.<br>Perching (resting on a narrow or elevated surface such as an artificial branch, log, or ledge).<br>Curling Up (contraction of the body into a compact or curled position, often with limbs tucked close to the torso).<br>Resting with Eyes Closed (closing the eyes while in a resting posture, indicating reduced alertness or sleepiness). All gestures when resting are not affected by environmental changes.
	Auto-grooming	AU	Individual inspection of various parts of the body using hands or mouth.
	Mobility	M	Walking (moving on the ground using all four limbs in a coordinated manner).<br>Running (moving rapidly on the ground with a bounding gait, often used for escape or pursuit).<br>Galloping (moving in a series of leaps or bounds, typically at high speeds).<br>Brachiation (swinging from branch to branch or fences using the arms while suspended beneath branches/fences).<br>Climbing (ascending or descending vertical surfaces, such as the fences, using all four limbs).<br>Leaping (jumping between vines).<br>Hanging (suspending the body from an artificial branch/fence or support structure using the arms or feet).<br>Swinging (propelling the body forward or sideways while suspended from a branch, vine, or rope).<br>Mobility while investigating the environment through exploration, navigation, spatial orientation behaviors, and exploratory movements that may include sniffing, touching, or visually scanning the surroundings.
	Stereotype	ST	Pacing (the individual moves back and forth repetitively within its enclosure). It may occur along a specific path or in a circular pattern.<br>Rocking (the individual sways its body rhythmically from side to side or back and forth while seated or standing).<br>Self-Injurious Behavior (the individual engages in actions that result in self-harm, such as biting, hitting, or scratching itself). It may lead to wounds, abrasions, or hair loss.<br>Bar Biting (the individual repetitively bites or gnaws on the bars or mesh of its enclosure). It may result in dental wear, damage to the enclosure, or injury to the primate.

			Hair Pulling (the individual plucks or pulls out its own fur or hair, either manually or using their teeth). It may lead to bald patches, skin irritation, or self-inflicted injuries. Over-grooming (the individual excessively grooms itself, focusing on specific body parts for prolonged periods). It may result in fur loss, skin lesions, or repetitive grooming injuries. Stereotypic Locomotion (the individual engages in repetitive locomotor patterns, such as pacing, spinning, or jumping in place). Head Bobbing (the individual repeatedly moves its head up and down or side to side rhythmically). Tongue Flicking (the individual flicks its tongue in and out of its mouth repetitively, often rapidly). Environmental Manipulation (the individual exhibits repetitive actions involving objects or environmental features within its enclosure, such as banging on surfaces, flipping objects, or rearranging bedding material). Vocalization Stereotypies (the individual emits repetitive vocalizations, such as grunts, screams, or whimpers, often in a rhythmic or patterned sequence).
Social	Affiliative	AF	Allo-grooming (mutual grooming between two or more individuals, involving the removal of parasites, debris, or dead skin from fur). Social touching (physical contact between individuals, such as touching, huddling, or embracing). Play Behavior (engaging in playful interactions, such as chasing, wrestling, or play fighting). Affiliative Vocalizations (emitting soft cooing, grunting, or chirping vocalizations during social interactions). Coalitions (forming temporary alliances or partnerships between individuals to achieve common goals, such as access to resources or defense against rivals). Proximity Maintenance (maintaining close physical proximity to preferred social partners or group members). Food Sharing (sharing food resources with conspecifics through cooperative interactions, such as offering, begging, or sharing). Affiliative Gestures (performing non-verbal gestures or signals to express affiliation, such as presenting, nodding, or patting). Social Grooming (engaging in grooming interactions with preferred social partners, either reciprocally or unidirectionally). Contact sitting (resting or sitting in close physical contact with preferred social partners or group members).
	Sexual	SE	Mounting (one individual climb on the back of another, often gripping the flanks or shoulders with its hands or feet). Copulation (intercourse between a sexually receptive female and a male, typically involving pelvic thrusting and genital contact). Genital display (presenting the genital region to potential mates through postural displays or movements). Solicitation (initiating sexual interactions through specific behaviors or vocalizations, such as presenting, vocalizing, or approaching potential mates and shows readiness to engage in mating activities).
	Agonistic	AG	Threat displays (displaying aggressive body postures or facial expressions, such as raised hackles, bared teeth, or staring).

	Vocalizations (emitting vocal signals associated with aggression, such as growling, barking, or screaming).<br>Charging (rapidly approaching an opponent with aggressive intent, often accompanied by vocalizations or exaggerated movements).<br>Chasing (pursuing a fleeing or subordinate individual with the intent to assert dominance or establish social hierarchy).<br>Physical aggression (engaging in physical combat or aggressive interactions, such as biting, scratching, or striking).<br>Submission (displaying submissive behaviors in response to threats or displays of aggression from dominant individuals).<br>Displacement (expelling subordinate individuals from preferred resources or social positions through aggressive interactions).<br>Staring (maintaining prolonged eye contact with an opponent as a form of threat or dominance display).<br>Vocal retaliation (responding to aggression or threat displays from conspecifics with vocalizations or aggressive vocalizations).<br>Avoidance (moving away from potential threats or disturbances, such as predators, conspecific aggression, or adverse environmental conditions).

28. Sha, J.C.M.; Hanya, G. Diet, activity, habitat use, and ranging of two neighbouring groups of food-enhanced long-tailed macaques (*Macaca fascicularis*). *Am. J. Primatol.* **2013**, *75*, 581–592. https://doi.org/10.1002/ajp.22137.

Analysis Set S1. Normality test for each behavioral data point.

1. Feeding
   Normality Test
   Shapiro-Wilk normality test

   data: ujianova.feeding_res
   W = 0.97179, p-value = 0.03624

   Homogeneity Test
   Levene's Test for Homogeneity of Variance (center = median)
   Df F value Pr(>F)
   group 7 2.0435 0.05831 .
   88

   Significant. codes: 0 '***' 0.001 '**' 0.01 '*' 0.05 '.' 0.1 ' ' 1

2. Moving
   Normality Test
   Shapiro-Wilk normality test

   data: ujianova.moving_res
   W = 0.92493, p-value = 3.71e-05

   Homogeneity Test
   Levene's Test for Homogeneity of Variance (center = median)
   Df F value Pr(>F)
   group 7 2.5394 0.01987 *
   88

   Significant. codes: 0 '***' 0.001 '**' 0.01 '*' 0.05 '.' 0.1 ' ' 1

3. Sexual
   Normality Test
   Shapiro-Wilk normality test

   data: ujianova.seksual_res
   W = 0.88459, p-value = 4.453e-07

   Homogeneity Test
   Levene's Test for Homogeneity of Variance (center = median)
   Df F value Pr(>F)
   group 7 1.2473 0.286
   88

4. Aggressive
   Normality Test
   Shapiro-Wilk normality test

   data: ujianova.aggressive_res
   W = 0.92115, p-value = 2.337e-05

   Homogeneity Test
   Levene's Test for Homogeneity of Variance (center = median)
   Df F value Pr(>F)
   group 7 1.7257 0.1132
   88

5. Stereotype
   Normality Test
   Shapiro-Wilk normality test

   data: ujianova.stereotipe_res
   W = 0.78618, p-value = 1.701e-10

   Homogeneity Test
   Levene's Test for Homogeneity of Variance (center = median)
   Df F value Pr(>F)
   group 7 6.1294 7.57e-06 ***
   88

   Significant. codes: 0 '***' 0.001 '**' 0.01 '*' 0.05 '.' 0.1 ' ' 1

Data Set S1. Body weight data of long-tailed macaques before and after the study.

No	Tattoo	SEX	Cage	BW	
				01/01/2023	01/04/2023
1	140416	F	K1	3,33	4,55
2	170708A	F	K1	2,59	2,78
3	C6446	F	K1	3,89	3,29
4	C7024	F	K1	4,36	3,53
5	C7397	F	K1	2,37	2,54
6	C7464	F	K1	2,95	4,01
7	J160609A	M	K1	5,57	5,52
8	N-AA0380	F	K1	2,48	3,35
9	N-FA1230	F	K1	2,52	3,15
10	N-FA2100	F	K1	3,06	4,6
11	N-FA2424	F	K1	2,32	3,95
12	N-FA3900	F	K1	2,68	3,64
13	N-FA4285	F	K1	2,71	2,71
14	N-FA4576	F	K1	2,4	3,38
15	N-FB1455	F	K1	2,84	3,68
16	N-FD0443	F	K1	2,15	2,5
17	T3974	F	K1	2,71	2,96
18	T4022	F	K1	2,86	3,42
19	221125	F	K1	2,84	3,67
	average			2,98	3,54

No	Tattoo	SEX	Cage	BW	
				01/01/2023	01/04/2023
1	130629	F	K2	3,06	2,95
2	130706	F	K2	2,99	4,28
3	130815	F	K2	3,13	3,75
4	140818A	F	K2	3,19	3,85
5	160823	F	K2	3,29	2,96
6	C1030	F	K2	3,83	4,97
7	IA3271	F	K2	3,16	2,27
8	I-FC9911	F	K2	2,72	2,15
9	J020908B	F	K2	2,37	2,76
10	J180808A	F	K2	2,6	2,79
11	J250307A	M	K2	4,49	4,98
12	T3724A	F	K2	3,45	3,45
13	T3966	F	K2	3,04	2,64
	Average			3,18	3,37

F = Female M = Male

Analysis Set S2. Normality test, Mann-Whitney test, and ANOVA analysis of the body weight of long-tailed macaques.

Body weight K1 group

(Nonparametric test using Mann Whitney test)

Normality Test

Shapiro-Wilk normality test

data: ujianova.bw1_res
W = 0.87098, p-value = 0.000426

Homogeneity Test

Levene's Test for Homogeneity of Variance (center = median)
Df F value Pr(>F)
group 1 0.0694 0.7937
36

Mann Whitney Test

Wilcoxon rank sum test with continuity correction

data: data.b1$jan and data.b1$apr
W = 87, p-value = 0.00661
alternative hypothesis: true location shift is not equal to 0

This proves a significant difference in MEP body weight before and after the provision of enrichment equipment based on the Mann Whitney Test (0.00661<0.05)

======================

Body weight K2 group

(Parametric test using One Way ANOVA)

Df Sum Sq Mean Sq F value Pr(>F)
treatment 1 0.237 0.2366 0.401 0.532
Residuals 24 14.146 0.5894

This proves that there is no significant difference in MEP body weight.

Analysis Set S3. Three-way ANOVA and Kruskal Wallis test for time, treatment, and diet type.

Three Way ANOVA

No	Behavior	p-value	Information
1	Eating	0.43927	Not significantly different
2	Resting	0.1049	Not significantly different
3	Affiliate	0.95664	Not significantly different
4	Auto grooming	0.266214	Not significantly different
5	Sexual	0.9804	Not significantly different
6	Agonistic	0.4042	Not significantly different

1. Eating
   Df Sum Sq Mean Sq F value Pr(>F)
   time 1 48.7 48.7 3.458 0.06681 .
   treatment 3 2562.2 854.1 60,583 < 2e-16 ***
   types of diets 2 572.3 286.2 20.298 8.65e-08 ***
   time:treatment 3 29.7 9.9 0.701 0.55408
   time:types of diets 2 152.0 76.0 5.390 0.00647 **
   treatment:types of diets 4 41.5 10.4 0.736 0.57041
   time:treatment:types of diets 4 53.6 13.4 0.951 0.43927
   Residuals 76 1071.4 14.1

   Significant. codes: 0 '***' 0.001 '**' 0.01 '*' 0.05 '.' 0.1 ' ' 1

2. Resting
   Df Sum Sq Mean Sq F value Pr(>F)
   time 1 2 2.4 0.223 0.6383
   treatment 3 4788 1595.8 145.885 <2e-16 ***
   types of diets 2 56 28.0 2.559 0.0841 .
   time:treatment 3 103 34.3 3.132 0.0304 *
   time:types of diets 2 101 50.4 4.608 0.0129 *
   treatment:types of diets 4 21 5.2 0.473 0.7557
   time:treatment:types of diets 4 87 21.7 1.987 0.1049
   Residuals 76 831 10.9

   Significant. codes: 0 '***' 0.001 '**' 0.01 '*' 0.05 '.' 0.1 ' ' 1

3. Affiliate
   Df Sum Sq Mean Sq F value Pr(>F)
   time 1 177.7 177.67 11.807 0.00096 ***
   treatment 3 122.9 40.96 2.722 0.05021 .
   types of diets 2 63.1 31.53 2.095 0.13012
   time:treatment 3 117.8 39.25 2.608 0.05765 .
   time:types of diets 2 14.1 7.03 0.467 0.62849
   treatment:types of diets 4 26.9 6.72 0.447 0.77440
   time:treatment:types of diets 4 9.8 2.45 0.163 0.95664
   Residuals 76 1143.7 15.05

   Significant. codes: 0 '***' 0.001 '**' 0.01 '*' 0.05 '.' 0.1 ' ' 1

4. Auto grooming
Df Sum Sq Mean Sq F value Pr(>F)
time 1 4.55 4.553 1.209 0.274968
treatment 3 82.52 27.507 7.305 0.000228 ***
types of diets 2 27.75 13.873 3.684 0.029700 *
time:treatment 3 18.14 6.046 1.606 0.195055
time:types of diets 2 6.70 3.348 0.889 0.415209
treatment:types of diets 4 18.28 4.569 1.213 0.312155
time:treatment:types of diets 4 20.04 5.011 1.331 0.266214
Residuals 76 286.16 3.765

Significant. codes: 0 '***' 0.001 '**' 0.01 '*' 0.05 '.' 0.1 ' ' 1

5. Sexual
Df Sum Sq Mean Sq F value Pr(>F)
time 1 0.704 0.7040 5.132 0.0263 *
treatment 3 6,682 2,2274 16,239 3e-08 ***
types of diets 2 0.190 0.0950 0.692 0.5035
time:treatment 3 0.205 0.0682 0.498 0.6851
time:types of diets 2 0.250 0.1249 0.910 0.4067
treatment:types of diets 4 0.450 0.1126 0.821 0.5158
time:treatment:types of diets 4 0.058 0.0144 0.105 0.9804
Residuals 76 10.425 0.1372

6. Agonistic
Df Sum Sq Mean Sq F value Pr(>F)
time 1 1.137 1.137 4.757 0.0324 *
treatment 3 14,972 4,991 20,878 6.67e-10 ***
types of diets 2 0.332 0.166 0.694 0.5030
time:treatment 3 1.303 0.434 1.816 0.1516
time:types of diets 2 0.188 0.094 0.393 0.6767
treatment:types of diets 4 1.618 0.404 1.692 0.1609
time:treatment:types of diets 4 0.973 0.243 1.017 0.4042
Residuals 74 17.689 0.239

Kruskal-Wallis (Non-parametric test)

No	Behavior	p-value	Information
1	Mobility	2.119e-05	Real different
2	Stereotype	0.001185	Real different

1. Mobility
Kruskal-Wallis rank sum test
data: data.mobility_treatment
Kruskal-Wallis chi-squared = 55.262, df = 19, p-value = 2.119e-05

2. Stereotype
Kruskal-Wallis rank sum test
data: data.treatment_stereotype
Kruskal-Wallis chi-squared = 43.286, df = 19, p-value = 0.001185
